# Risk factors for mediastinitis and mortality in pneumomediastinum

**DOI:** 10.34172/jcvtr.2022.09

**Published:** 2022-03-15

**Authors:** Hülya Dirol, Hakan Keskin

**Affiliations:** ^1^Department of Thoracic Disease, School of Medicine, Akdeniz University, Antalya, Turkey; ^2^Department of Thoracic Surgery, School of Medicine, Akdeniz University, Antalya, Turkey

**Keywords:** Pneumomediastinum, Mediastinal Emphysema, Iatrogenic, Pneumomediastinum

## Abstract

***Introduction:*** Pneumomediastinum (PM) is a self-limiting disease with a good prognosis. Mediastinitis is a rare but potentially fatal complication of PM. Identification of risk factors for mediastinitis is essential for better management.

***Methods:*** This is a single-center, retrospective study conducted in a university hospital. Adult patients with PM between January 2016 and June 2020 were involved in the study. The data about age, gender, symptoms, signs, treatment, development of mediastinitis, hospital stay, and mortality were investigated.

***Results:*** In total, 79 patients with PM were analyzed. The most common symptom was dyspnea(58;73.4%) and the most common sign was subcutaneous emphysema (48;60.7%). Thirty(37.9%) of them were iatrogenic PM (IPM), while 22 (27.9%) were spontaneous PM (SPM) and27 (34.2%) were traumatic PM (TPM). Mediastinitis developed in 17 (12 from IPM, 4 from TPM,1 from SPM) patients, and 11 (58.8%) of these patients died. The incidence of mediastinitis in the IPM group was significantly higher than in the TPM and SPM group (respectively, *P* = 0,03,*P* = 0,01). There was no significant difference between the age, gender, symptoms, and signs of those with or without mediastinitis. Mortality was lower in TPM and SPM than IPM (respectively,*P* = 0,05, *P* = 0,03), and hematological malignancy was remarkably common in patients who died from mediastinitis in the TPM and SPM group.

***Conclusion:*** Mediastinitis and mortality were significantly higher in IPM, while hematological malignancy was remarkably prevalent in patients deceased from mediastinitis in TPM and SPM.

## Introduction

Pneumomediastinum, the presence of air in the mediastinum was first described by Laennec in 1819 in a 4-year-old boy who was run over by a manure cart. It has also been described as mediastinal emphysema.^1-3^ Although pneumomediastinum had developed as a result of trauma to the thorax in the first patient, it was understood that it may be associated with many other situations besides trauma.

Based on differences in the etiology pneumomediastinum is divided into primary and secondary. Primary pneumomediastinum is a spontaneous pneumomediastinum that develops without any invasive procedure or trauma. Secondary pneumomediastinum is divided into two as traumatic pneumomediastinum which is secondary to trauma and iatrogenic pneumomediastinum secondary to invasive procedures.

Mediastinitis, which is an infrequent but major complication of pneumomediastinum should be suspected in patients with fever, elevated leukocyte count, C-reactive protein or sedimentation rate. It is important to determine this complication as possible as early to prevent poor outcome. Here, in this study, we evaluated the characteristic features of the patients with pneumomediastinum and investigated the risk factors for the development of mediastinitis and mortality in pneumomediastinum.

## Material and Methods

Adult patients with PM between January 2016 and June 2020 were retrospectively analyzed. Routinely we performed chest tomography for all patients with trauma. In cases of clinical suspicion or a finding indicating pneumomediastinum on the chest x-ray in non-traumatic patients, we also performed chest tomography. Chest x-rays of 12 patients in the iatrogenic group and 9 patients in the spontaneous group had signs of pneumomediastinum. The presence of pneumomediastinum was proved by thoracic tomography in all these patients. In this study, all the patients Only patients whose diagnosis was finalized by thoracic tomography were included in this study.

Patients with PM were divided into 3 groups as traumatic, iatrogenic, and spontaneous. Patients with PM after any trauma were included in the TPM group. Patients in whom PM developed as a result of any medical intervention other than thoracotomy and sternotomy were included in the IPM group. Patients without trauma or medical intervention were also included in the SPM group.

Age, gender, symptoms (shortness of breath, chest pain, cough, dysphagia) and signs (subcutaneous emphysema, neck swelling), treatment procedures (prophylactic antibiotic use, chest tube), complications, hospital stay, mortality at hospital, and the medical intervention data were collected through a protocol. If the patient had a fever, elevation in leukocyte count, C reactive protein, and sedimentation during the hospital stay, we diagnosed mediastinitis. We investigated the risk factors for mediastinitis. Ethical approval for this study was obtained from Akdeniz University Medical Faculty Clinical Research Ethics Committee. (No: KAEK-897).

### Statistical analysis

IBM SPSS Statistics Version 23 program was used for statistical analysis of the findings obtained from the study. Categorical variables are expressed as percentages and continuous variables are reported as mean ± standard deviation (SD). Chi-square test or Fisher’s exact test was used for categorical variables. The patients were divided into three groups (iatrogenic, trauma, and spontaneous) and analyzed by one-way analysis of variance (ANOVA) and a linear trend test was performed. *P* < 0.05 values were considered to be statistically significant.

## Results

### Characteristics of the patients with pneumomediastinum

A total of 87 patients older than 18 years of age were diagnosed with PM through chest computed tomography (CT) between January 2016 and June 2020 in our hospital. Three patients with PM developed after sternotomy, 4 patients with PM developed after thoracotomy, and 1 patient with PM who refused hospitalization were excluded.

Of the 79 patients included in the study, 69 (87.3%) were male and the mean age was 50.1 ± 19.1 years. The most common symptom was dyspnea (58; 73.4%) while the most common sign was subcutaneous emphysema (48; 60.7%). Extrapleural air sign (31 %35,6) was the most common chest x-ray finding. Thirty (37.9%) patients were in the IPM group, while 22 (27.9%) patients in SPM and 27 (34.2%) patients in the TPM group. The characteristics of all three groups are summarized in Table 1.

**Table 1:** The characteristics of the patients according to pneumomediastinum subgroups.

		**İatrogenic**	**Traumatic**	**Spontaneous**	***P*** ** value**
Age		60.7 ± 15.8	37.8 ± 14.8	50.6 ± 13.2	0.01
Gender	Female	7	0	3	0.02
	Male	23	27	19	
Chest pain		17	25	15	0.01
Dyspnea		19	21	18	0.27
Cough		13	4	13	0.01
Dysphagea		9	3	0	0.01
Subcutaneous emphysema		17	18	13	0.73
Neck swelling		15	12	8	0.61
Pneumothorax		10	21	9	0.01
Chest tube		9	19	8	0.01
Esophagoscopy &Bronchoscopy		2	0	1	0.42
Antibiotics		25	26	17	0.14
Mediastinitis		12	4	1	0.01
Hospital Stay		22.1 ± 13.9	24.5 ± 14.6	13.4 ± 7.9	0.02
Mortality at hospital		8	2	1	0.03

### Patients with Iatrogenic Pneumomediastinum

We analysed 30 patients with iatrogenic pneumomediastinum. The most common invasive procedure causing iatrogenic pneumomediastinum was the abdominal surgery. Tube thoracostomy and bronchoscopy/esophagoscopy were the other leading procedures (Table 2). Nine (30%) patients in this group had PM-related dysphagia. The frequency of dysphagia in iatrogenic pneumomediastinums was significantly higher than in the spontaneous pneumomediastinum group (*P* = 0.01). Mediastinitis developed in 12 (40%) of the patients in iatrogenic pneumomediastinum group. It developed more frequently after endoscopy (66.7%). Mediatinitis was significantly more common in iatrogenic pneumomediastinum group than in the spontaneous pneumomediastinum group (*P* = 0.01). Eigth (26.7%) patients died in the iatrogenic pneumomediastinum group and mortality rate was significantly higher in the iatrogenic pneumomediastinum group compared to the spontaneous pneumomediastinum group (*P* = 0.03).

**Table 2:** The distribution of the procedures in iatrogenic pneumomediastinum.

	**Number (n)**	**Percent (%)**
Abdominal surgery	7	23.3
Tube Thoracostomy	6	20
Bronchoscopy or Esophagoscopy	6	20
Tracheostomy or neck surgery	5	16.7
Vertebral surgery	4	13.3
Intubation or mechanical ventilation	2	6.7

### Patients with Traumatic Pneumomediastinum

Twenty-seven (34.2%) patients were in the traumatic pneumomediastinum group. The mean age of the patients in this group was 37,8 ± 14,8 years which was significantly lower than the mean age of the patients in iatrogenic pneumomediastinum and spontaneous pneumomediastinum group (*P* = 0.01, *P* = 0.01, respectively). There was no female in the traumatic pneumomediastinum group. The most common symptom in this group was chest pain (92.6%). Cough was significantly lower (*P* = 0.01, *P* = 0.01, respectively) while the chest pain was significantly higher (*P* = 0.01, *P* = 0.01, respectively) in traumatic pneumomediastinum group than in the iatrogenic pneumomediastinum and spontaneous pneumomediastinum group. Pneumothorax (21 (77.7%) patients) and tube thoracostomy (19 (70.4%) patients) were significantly more common in the traumatic pneumomediastinum group than in iatrogenic pneumomediastinum and spontaneous pneumomediastinum group (*P* = 0.01, *P* = 0.01, respectively).

### Patients with Spontaneous Pneumomediastinum

Twenty-two (27.9%) patients were in the spontaneous pneumomediastinum group. The mean age of the patients was 66.58 years. One patient (4.5%) died in this group. The patients were younger (*P* = 0.02) and the mortality rate was lower (*P* = 0.03) in the spontaneous pneumomediastinum group than in the iatrogenic pneumomediastinum group. The hospitalization stay length (13.4 ± 7.9 days) was shorter in the spontaneous pneumomediastinum group than in iatrogenic pneumomediastinum and spontaneous pneumomediastinum group (*P* = 0.01,*P* = 0.01, respectively).

### Risk factors for mediastinitis and mortality

Mediastinitis developed in 17 (21.5%) of all pneumomediastinum patients and of these, 11 (58.8%) patients deceased. There was no significant difference in age, gender and symptoms between patients with mediastinitis and those who did not (Table 3). But there was a significant difference in the frequency of mediastinitis according to the etiology. Mediastinitis was more common in iatrogenic pneumomediastinum than in traumatic and spontaneous pneumomediastinum (*P* = 0.03, *P* = 0,01, respectively).

**Table 3:** The comparison between patients with and without mediastinitis.

		**Medistinitis**		
		**No**	**Yes**	***P*** ** value**
Age		48 ± 19.01	55.19 ± 18.71	0.11
Gender	Female	9	1	0.34
	Male	53	16	
Chest pain		46	11	0.44
Dyspnea		47	11	0.38
Dysphagea		7	5	0.7
Cough		26	4	0.16
Hospital stay		17.70 ± 13.71	31.69 ± 12.67	0.01
Mortality at hospital		1	10	0.01

Twelve (40%) patients from iatrogenic pneumomediastinum group had mediastinits and 8 (66.6%) of them deceased while 1 (4.5%) patient from spontaneous pneumomediastinum group had mediastinitis and this patient who had a hematological malignancy, deceased. 4 (14.8%) patients from traumatic pneumomediastinum group had mediastinitis and 2 of them deceased and one of the decased patients also had a hematological malignancy. Mortality was significantly more common in patients with mediastinitis than those without mediastinitis (p = 0.01) (Table 4). Moreover, mortality was significantly more common in iatrogenic pneumomediastinum group than in traumatic and spontaneous pneumomediastinum group (*P* = 0,05, *P* = 0,03, respectively).

**Table 4:** The comparison between survivors and nonsurvivors.

		**Nonsurvivor**	**Survivor**	***P*** ** value**
Age		56.91 ± 17.10	48.99 ± 1920	0.34
Gender	Female	0	11	0.34
	Male	11	58	
Chest pain		7	50	0.49
Dyspnea		8	50	0.06
Cough		3	27	0.43
Dysphagea		2	10	0.76
Subcutaneous emphysema		7	41	0.83
Neck swelling		8	27	0.05
Pneumothorax		5	35	0.71
Chest tube		4	32	0.50
Esophagoscopy		0	9	0.34
Bronchoscopy		1	2	0.32
Antibiotics		9	59	0.66
Mediastinitis		10	7	0.01
Hospital stay		26.64 ± 13.70	19.54 ± 14.58	0.07

## Discussion

In this study, we observed that mediastinitis and mortality were significantly higher in iatrogenic pneumomediastinum compared to the others. Age, gender, and symptoms do not appear to have an effect on the development of mediastinitis. Mortality was associated with the development of mediastinitis. There was a significant difference between the length of hospital stay and mortality between those who developed mediastinitis and those who did not. Moreover, hematological malignancy may be important in terms of mortality associated with mediastinitis in traumatic and spontaneous pneumomediastinum. Furthermore, hematological malignancy was a remarkable feature of the patients who died from mediastinitis in the traumatic and spontaneous pneumomediastinum group.

Iatrogenic pneumomediastinum was the most common among all pneumomediastinum subgroups in this study. Iatrogenic pneumomediastinum can occur secondary to any invasive procedure in head and neck. The fascial planes in the neck communicate with the mediastinum via the retropharyngeal space. Similarly, there is a communication between the chest and abdomen facial planes.^4,5^ Abdominal surgeries were the most common procedure causing iatrogenic pneumomediastinum in this study. The first harbinger of an anastomotic leakage after abdominal surgery may be the symptoms and signs associated with pneumomediastinum.^6,7^ Therefore, surgeons who perform head, neck and abdominal surgeries should be familiar with the symptoms and signs of pneumomediastinum.

Dyspnea, cough, chest pain and dysphagia are the main symptoms of pneumomediastinum. None of these symptoms seem to predict the development of mediastinitis or mortality. There was no difference in these symptoms between survivors and nonsurvivors or patients who had mediastinitis and those who did not. But there were significant differences in symptom distribution among the pneumomediastinum subgroups. While dyspnea was observed with a similar frequency in all three groups, chest pain was more common in traumatic pneumomediastinum, cough and dysphagia were more common in spontaneous pneumomediastinum. Pneumothorax was more common in the traumatic pneumomediastinum group, so there was more chest tube application also. But, neither mediastinitis nor mortality was associated with the development of pneumothorax and the chest tube application.

Major airway and esophageal injuries accompanying pneumomediastinum are possible in thoracic trauma. ^8-11^ Airway or esophageal injuries may occur in both penetrating and blunt trauma. The small size and the relatively preserved location of these structures might be the reason of why it so rarely accompanies the pneumomediastinum.^12^ There was no eosophageal or airway injury in patients with traumatic pneumomediastinum in our study. This may have contributed to the low mortality in this group.

Previously, Mansella and collageous found in their study that traumatic pneumomediastinum was more common in younger. Then, they suggested that increasing stiffness of the pulmonary interstitium by aging might preserve elders from the development of traumatic pneumomediastinum.^13^ Incidence might be lower in elders but the outcome absolutely is worse in elders when it happens. David et al suggested that mediastinal structures are more fragile with increasing age and so the traumatic pneumomediastinum is more serious in elders.^14^ In our study, all patients in the traumatic pneumomediastinum group were younger than 65 years old. This may have contributed to the better outcome in traumatic pneumomediastinum also.

For the evaluation of airway or eosophageal injuries, bronchoscopy or esophagoscopy can be performed. Most of the main airway injuries can be managed without surgery. However, the outcome of esophageal injuries may be poor if diagnosis or surgical intervention is delayed.^11^ An accompanying esophageal injury, that is asymptomatic and insidious, distress chest surgeons as the late detection of esophageal rupture is associated with high mortality.^11^ Nevertheless, the necessity of aggressive approaches such as a routine esophagoscopy or bronchoscopy for evaluation of the aerodigestive system in patients with pneumomediastinum is controversial. Some recommend a more conservative approach in the absence of esophageal rupture signs in chest computerized tomography.^8,11^ In our study, we did not perform bronchoscopy and esophagoscopy aggressively. There was no difference in terms of incidence of these procedures application between the pneumomediastinum subgroups. Among the patients for whom we performed, esophageal perforation was detected in only one patient. Moreover, pneumomediastinum was complicated with mediastinitis in this patient who is still under follow-up due to esophageal stenosis. There was no difference in the frequency of esophagoscopy and bronchoscopy between the survivors and nonsurvivors or between pneumomediastinum subgroups or between the patients who had mediastinitis and those who did not. So, we suggest to being conservative in terms of bronchoscopy or eosophagoscopy and perform these in only cases with high clinical or radiological suspicion.

There are many well-managed spontaneous pneumomediastinum case series, with only a few reporting poor outcomes.^15^ It usually occurs in young adults and exerts a self-limiting and benign course.^16^ Recently, some authors suggested that these patients can be managed on an outpatient basis. Takeda et al suggested that a 2-day follow-up is feasible.^17^ In a recent study, 23 of 34 patients with spontaneous pneumomediastinum were followed up as an outpatient manner, only two, were given prophylactic antibiotics, and none of 34 patients developed any mediastinitis.^16^ In our study, there was only 1 patient with mediastinitis in this group and this patient did not survive. This patient had hematological malignancy. Probably, this has contributed this poor outcome. Apart from such comorbidity, there is a low risk for the development of mediastinitis and mortality in spontaneous pneumomediastinum.

In our study, we found that the patients were in high risk for the development of mediastinitis and mortality if the pneumomediastinum arised secondary to invasive procedures. This may be related with the underlying diseases of these patients. Additionally, the patients in this group were also older than the others. Thus the outcome was poor in patients with iatrogenic pneumomediastinum. The mediastinitis developed more frequently after endoscopy in our study. Moreover, there was no mediastinitis or death in vertebral surgery-related pneumomediastinum. So, the type of the invasive procedure may also affect the outcome.

Our study has some limitations. The small number was the major limitation. The number of cases that we were able to collect as a single center in approximately 5 years was only this much. Since the study was retrospective, not every data was available for every patient, some data were missing. In order to determine risk factors for mediastinitis and mortality, it is essential to design a prospective multicenter study in accordance with protocols with specific primary and secondary endpoints.

## Conclusion

Although pneumomediastinum is generally a self-limiting disease with a good prognosis, we should be more careful about the development of mediastinitis in patients with iatrogenic pneumomediastinum. Iatrogenic pneumomediastinum can be a life-threatening complication. In our study, we found that the risk of mortality in iatrogenic pneumomediastinum was higher than in traumatic and spontaneous pneumomediastinum. Mortality was associated with the development of mediastinitis. The patients with iatrogenic pneumomediastinum had mediastinitis more frequently. The reason for the increased mortality in iatrogenic pneumomediastinum was increased frequency of mediastinitis. When we examined the factors that may be associated with the development of mediastinitis, we could not find any relationship between the development of mediastinitis and age, gender, symptoms and findings. We think that the underlying comorbidities of the patients in the iatrogenic group may be responsible from the increased risk for mediastinitis.

## Acknowledgements

There are no acknowledgments to be mentioned in this study.

## Funding

None.

## Ethics approval

Ethical approval for this study was obtained from Akdeniz University Medical Faculty Clinical Research Ethics Committee. (Ethics number: KAEK-897).

## Competing intrests

The authors declare that they have not conflicts of interest.
